# Absorbent hygiene products disposal behaviour in informal settlements: identifying determinants and underlying mechanisms in Durban, South Africa

**DOI:** 10.1186/s12889-024-18396-y

**Published:** 2024-03-28

**Authors:** Jurgita Slekiene, Nick Swan, Marc Kalina

**Affiliations:** 1 Department of Consultation-Liaison Psychiatry and Psychosomatic Medicine, University Hospital Zurich, University of Zurich, Zurich, Switzerland; 2 Ranas Ltd, Zurich, Switzerland; 3 Green Corridors, Durban, South Africa; 4 Department of Mechanical and Process Engineering, ETH Zurich, Zurich, Switzerland; 5 School of Engineering, University of KwaZulu-Natal, Durban, South Africa

**Keywords:** Behaviour change, RANAS, Mental health, Absorbent Hygiene products (AHPs), South Africa

## Abstract

**Background:**

Within South Africa, many low-income communities lack reliable waste management services. Within these contexts, absorbent hygiene product (AHP) waste, including nappies (diapers), are not recycled, and are often dumped, ending up in watercourses and polluting the local environment. The structural barriers to collection which have been well explored, however the behavioural determinants of safe disposal for AHPs remains poorly understood. The purpose of this study is to determine the psycho-social factors driving AHP disposal behaviour for caregivers, while identifying potential underlying mechanisms (such as mental health), which may be influencing disposal behaviour, with the intention of informing a future, contextually appropriate and sustainable, collection system.

**Methods:**

The cross-sectional study was conducted within three low-income communities located within eThekwini Municipality (Durban), South Africa. The study included a pre-study and a quantitative survey of 452 caregivers, utilising the RANAS approach of behaviour change. The quantitative questionnaire was based on the RANAS model to measure psycho-social factors underlying sanitary disposal of AHPs. Mental health was assessed using the Self-Reporting Questionnaire (SRQ-20). Statistical analysis involved regressing psycho-social factors onto disposal behaviour and exploring their interaction with mental health through a moderation model.

**Results:**

Our findings suggest that one third of caregivers do not dispose of nappies sanitarily, despite intent (86.9%). Regression analysis revealed ten psycho-social factors which significantly predict the desired behavioural outcome, the sanitary disposal of AHPs. Caregivers with poor mental health were less likely to dispose of AHP sanitarily, which reflects previous research linking poor mental health and the impairment of health-related daily activities, particularly within vulnerable groups. Specifically, several psycho-social factors underlying were moderated by poor mental health, the prevalence of sanitary disposal of AHPs depended on mental condition of caregiver.

**Conclusions:**

Our findings confirmed the link between poor mental health and unsanitary AHPs disposal. This is especially relevant because poor mental health is common within South Africa. Addressing mental health problems within these communities is an essential step to providing sustainable waste management services. The findings informed an intervention strategy to implement a future collection system for these communities, and similar low-income or informal contexts within South Africa.

**Supplementary Information:**

The online version contains supplementary material available at 10.1186/s12889-024-18396-y.

## Introduction

Across the globe, inequality underpins access to waste management systems, structuring who can or cannot utilise or provide sustainable services [1–3]. In South Africa, the most unequal country in the world [4], this is particularly the case, where nearly half the population lacks access to municipal waste collection [2, 5]. Although the democratic South African state has made great strides over the past two decades to extend service provision to previously un-serviced areas, this gap remains the most prominent in historically non-white communities, including traditionally governed rural and peri-urban land, as well as the multitude of informal settlements which have proliferated within, and on the margins of, South Africa’s cities [6, 7]. This inequality contributes to numerous health and safety impacts on affected communities, who are burdened with unclean spaces and riskier disposal options, while contributing to the leakage of solid waste into the natural environment, including our rivers and oceans (Kalina et al., 2022a). Within the City of Durban, located on South Africa’s eastern coast and part of the larger eThekwini Metropolitan Municipality, Municipal officials have embarked on efforts to ‘upgrade’ informal settlements, and provide basic services, including waste collection [9, 10]. However, financial constraints within the municipality, the logistical hurdles of providing services within informal spaces, and the inability for poor residents to pay, has severely hampered the provision of reliable waste management services [11].

Absorbent Hygiene Products (AHPs), which include disposable tissues, diapers, and feminine hygiene products, are essential to human dignity and hygiene, especially for women, the elderly, the ill, people who menstruate, parents, and other caregivers. Currently, few end-of-life (EoL) options exist for AHP waste, especially within the Global South, where the majority of AHP waste is disposed of within dumpsites or landfills [12]. Moreover, because the use of AHPs is expected to rise, it is anticipated that AHPs will become a growing waste management challenge, particularly in Southern cities, with less robust Municipal Solid Waste (MSW) systems, and which, as the ongoing Covid-19 pandemic has demonstrated, are less able to manage increases in potentially hazardous waste [13–15]. Within South Africa, AHP waste, including nappies (diapers), are not recycled, and are often dumped, especially in low-income communities (Schenck et al., 2019; Schenck et al., 2022). Moreover, the disposal of AHPs, and feminine hygiene products in particular, is complicated by taboo or stigma, which in many cultures, including within South Africa, is attached to menstrual blood, forcing women into often hidden or unsafe disposal pathways for these items, such as in the bush or down the toilet (Kalina et al., 2022b; Roxburgh et al., 2020). As a result, improperly disposed AHPs are a significant source of waste leakage into the natural environment, where in Durban, especially in low-income communities, AHP waste often litters hillsides and clogs storm water drains, from where it washes into our rivers, and eventually the sea (Kalina et al., 2022a). Given the challenges of providing solid waste management services and the difficult socio-economic conditions within these contexts, what drives AHP disposal behaviour, and what underlying mechanisms may be influencing individual disposal decisions?

Moreover, previous research from the World Health Organisation (WHO) [20] has suggested that underlying mechanisms, including chronic illness and mental health may be impacted by waste within the environment, while influencing waste management behaviours of affected individuals. Furthermore, previous research from within Southern Africa has suggested that poor mental health and depression can impair daily activities in vulnerable groups, including children and youth [21–23]. This connection is particularly relevant within South Africa, where the prevalence of mental disorders is particularly high (30.3%) [24].

The purpose of this study is to determine the psycho-social factors driving AHP disposal behaviour for mothers and caregivers, while identifying potential underlying mechanisms (such as mental health), which may be influencing disposal behaviour, with the intention of informing a future, contextually appropriate and sustainable, collection system. Although there has been some investigation of behaviour factors driving recycling within South Africa [25, 26], psycho-social evaluation has not yet, as far as we know, been utilised to investigate AHP disposal.

Specifically, we ask: (1) which psycho-social factors are determinants for sanitary disposal of AHPs among caregivers in low-income contexts, and (2) how does mental health influence caregivers’ sanitary disposal of AHPs? This work directly responds to a knowledge gap on the behavioural determinants of safe disposal and collection of AHPs, both in South Africa and globally. To identify the behavioural factors associated with caregiver’s sanitary disposal and collection of AHPs behaviour, this study utilised the Risks-, Attitudes-, Norms-, Abilities- and Self-regulation (RANAS) approach of behaviour change, a methodological approach for developing, implementing, and evaluating behaviour change (BC) strategies, that has been utilised in many South and low-income contexts similar to our case study [27–31].

## Methodology

### Study design, location and period

**Fig. 1:**
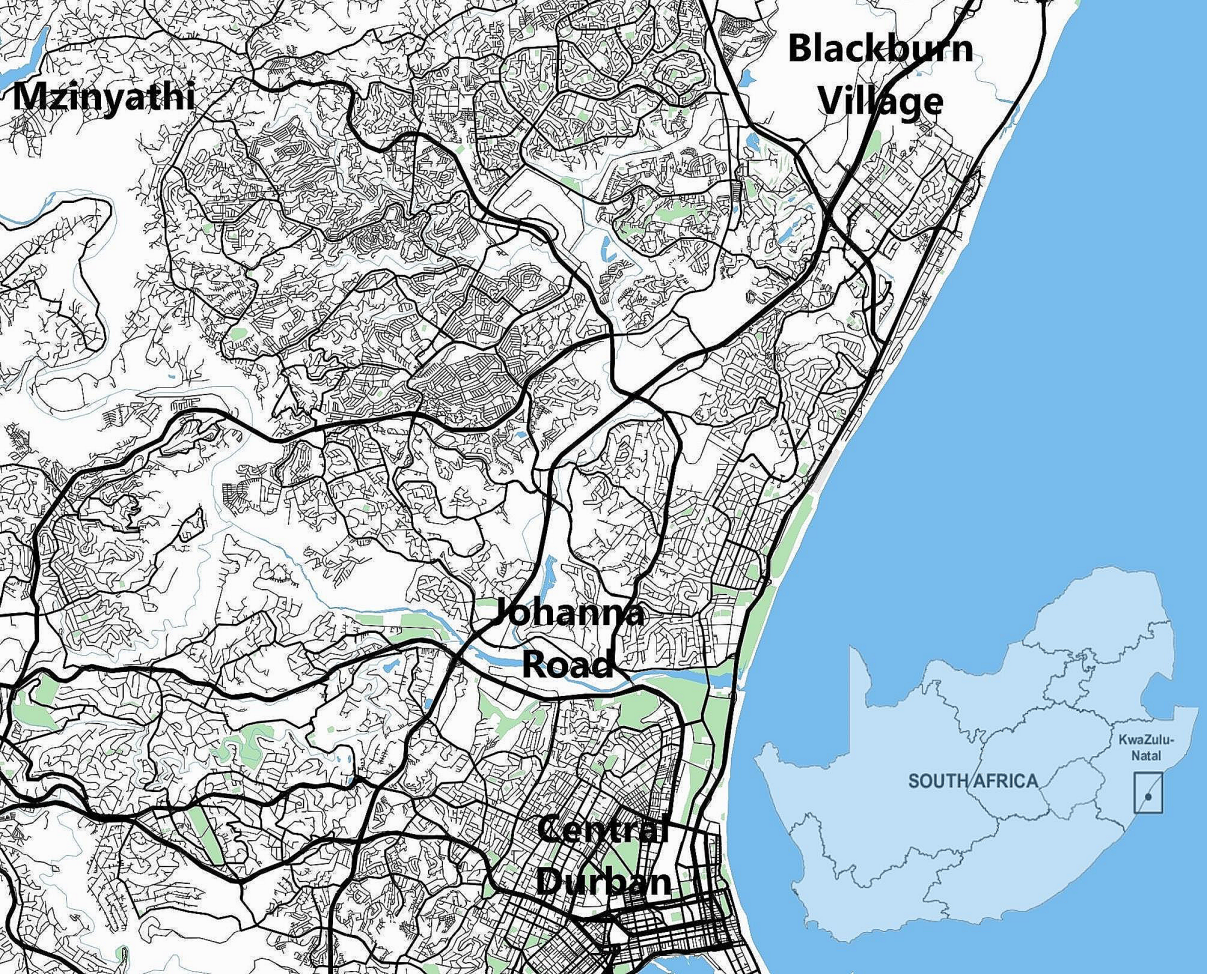
Community locations in relation to central Durban (Map Data Source: Mappin WMS)

This cross-sectional study included a qualitative pre-study and a quantitative survey in low-income communities related to Durban, South Africa. Data collection took place in three pre-selected low-income communities: Mzinyathi, Johanna Road Informal Settlement, and Blackburn Village in eThekwini Municipality from September to November 2022. Each are low-income settlements within eThekwini municipal boundaries (Fig. 1). Both Johanna Road and Blackburn Village are informal settlements, which are housing areas that have been illegally built on municipal land, giving the appearance of impermanence, but over time, have become established communities. Mzinyathi, by contrast, is a sprawling, peri-urban settlement on the fringe of the municipality. Although they share similarities, the communities are differentiated in terms of housing construction density, settlement size, accessibility from the developed urban commercial-industrial centres, and surrounding land use. However, all three suffer from a variety of service delivery challenges, as Johanna Road and Blackburn Village are informal, and do not receive regular municipal services, and Mzinyathi is located in traditional authority land, and likewise is not serviced by the municipality as residents do not pay rates. As a result, waste management is a significant challenge in all three communities, with an immense amount of waste leaking into the natural environment from improper disposal and a lack of collection. Moreover, all three are located within close proximity of natural watercourse within a few miles of the ocean, hence increasing the environmental beneficial impacts of the study.

### Study participants, sampling methods and sample size

The study participants were caregivers of children up to 5 years. The pre-study involved three focus group discussions (FGD’s, *N* = 30) with caregivers (all of them were mothers of a child up to five years). A pre-study was conducted to inform and develop the quantitative survey. The participants for the quantitative survey were selected using a random route method (every second house). In total, *N =* 452 caregivers were recruited for our research study. Participation in the study was completely voluntary. Written, informed consent was obtained from each participant. No individuals under the age of 18 were included and the study did not encounter illiterate participants.

### RANAS

**Fig. 2:**
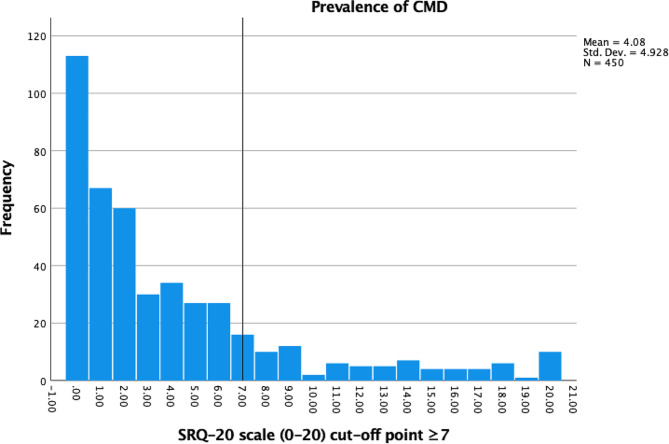
Prevalence of common mental disorders (CMD) among mothers and caregivers

The RANAS model has been developed using various psychological theories [32, 33]. The model consists of five psychosocial factor blocks. Risk factors include health related knowledge, perceived vulnerability, and perceived severity of the target behaviours. Attitude factors include beliefs about the costs and benefits of a target behaviour and feelings arising while performing the target behaviour. Norm factors comprise perceived social influence, such as behaviour of others, others’ approval, and personal importance. Ability factors include confidence in performance of a particular behaviour. Self-regulation factors cover management of conflicting goals and barriers, commitment, and remembering to perform the target behaviour. Furthermore, the RANAS model considers not only psycho-social factors underlying intention, habit and behaviour, but also three domains of contextual factors: social, personal, and physical contexts. Culture, social relations, laws and policies, economic conditions, and the information environment constitute the social context. The natural and built environments comprise the physical context. Age, gender, education, individual differences in the physical and mental health of the person and are part of the personal context (Fig. 2).

Figure 2 The Ranas model [33].

### Questionnaires and measures

The structured, face-to-face interviews were conducted in isiZulu. The quantitative questionnaire was based on the RANAS model. Most of the questions were closed, such as those about the target behaviour and the psychosocial factors underlying target behaviour. Questions were measured on 5-point scale [from ‘not at all’ to ‘very much’; from ‘at no time’ to ‘almost each time’; from ‘never’ to ‘very often’; from ‘nobody’ to ‘almost all of them’]. The SRQ-20, a 20-items screening instrument which was developed by WHO and widely used in low- and middle-income countries, was used to assess mental health among mothers and caregivers [34]. A research assistant in South Africa translated the questionnaire from English to isiZulu. Subsequently, a different research assistant in South Africa translated the questionnaire back to English from isiZulu to verify the precision of the translation. Moreover, during the eight days training of the local research assistants and enumerators’, detailed discussions were held to ensure a comprehensive understanding of the intent behind each question.

### Ethical, safety and regulatory issues

The study research protocol was approved by the Ethics Committee of the ETH Zurich in Switzerland [EK-2022-N-155] and the University of KwaZulu-Natal Research Ethics Committee [REC-040414-040]. All procedures applied in the research study were in accordance with the Declaration of Helsinki. All study participants were over the age of 18 and provided written informed consent. For those are unable to read or write, the consent statement was read aloud and individuals provided consent by making a mark on the subject signature line.Participants were provided with a unique identifying number, and data were anonymized during data analysis. Data were accessed only by the authors.

### Statistical analysis of data

The statistical analysis of data was conducted using IBM SPSS 28 Statistics software and the PROCESS macro for SPSS [35]. To identify the most influential behavioural determinants, psycho-social factors of the RANAS model underlying target behaviour (independent variables) were regressed onto the sanitary disposal AHPs as outcome (the dependent variable). Correlations were used to investigate associations between study variables such as sanitary disposal of AHPs, and mental health. T-tests and effect size calculations were used to compare means between poor and good mental health groups [36]. A regression analysis method, PROCESS (see [37]) was applied to calculate moderation model. The moderation model was used to test for interaction (when two variables influence each other’s effects). Our moderation model included mental health as the moderator (M), sanitary disposal of AHPs as the outcome (Y), and psycho-social factors as predictors (X). Only significant factors from linear regression analysis were tested in a moderation model. Moderation analysis was used to test the interaction between the moderator M (mental health) and predictors X (psycho-social factors) in a model with outcome Y (sanitary disposal of AHPs). With evidence that X’s effect is moderated by M, the analysis should confirm X’s effect on Y at various values of the moderator (Scale: 0–20 in our model).

## Results

### Characteristics of the RANAS sample

From total 452 caregivers, 90.7% were female (*N* = 410) and 9.3% male (*N* = 42). The age of study participants was categorised in six categories, 18–24 years old were 20.6% (*N* = 93), 25–29 years old were 38.9% (*N* = 176), 30–39 years old 26.8% (*N* = 121), 40–49 years old 8.2% (*N* = 37), 50–59 years old 3.3% (*N* = 15) and 2.2% (*N* = 10) were 60 years and more. The majority (86.3%) of caregivers were 18–39 years old at the time of assessment. Average household size among the study population was *M* = 4.27 (*SD* = 1.94) and children under two years in a household *M* = 1.40 (*SD* = 0.77) (other characteristics in Table 1).

**Table 1:** Sociodemographic Characteristics of Participants (n, %)

Characteristics	n	%
Gender		
Female	410	90.7
Male	42	9.3
Community		
Blackburn	143	31.6
Johanna Road	135	29.9
Mzinyathi	174	38.5
Marital status		
Married	53	11.7
Single	357	79.0
Cohabiting	32	7.1
Widow(er)	6	1.3
Divorced	4	0.9
Religion		
Christians	353	78.1
African traditional	59	13.1
Not religious	27	6.0
Other	13	2.9
Educational level		
None or don’t know	1	0.2
Can read but not write	1	0.2
Can read and write	12	2.7
Primary	44	9.7
Secondary	319	70.6
College and higher	73	16.2
University degree	2	0.4
Employment status		
Permanent employed	37	8.2
Part-time employed	57	12.6
Casual employment	38	8.4
Self employed	32	7.1
Unemployed	265	58.6
Other	23	5.1
Mobile phone ownership ^a^	395	87.4
Smartphone ^a^	293	64.8
Facebook ^a^	265	58.5
WhatsApp ^a^	294	65.0
Instagram ^a^	33	7.3
Twitter ^a^	8	1.8
Wealth Index		
TV ^a^	299	66.2
Radio ^a^	221	48.9
Bicycle ^a^	73	16.2
Running water ^a^	265	58.6
Electricity ^a^	429	94.9
Moto ^a^	69	15.3
Auto ^a^	37	8.2
Income per month		
Less than R1 000	239	52.9
R1 000– R2 499	127	28.1
R2 500– R4 999	62	13.7
R5 000– R10 000	18	4.0
R10 000 +	6	1.3

Note. *N* = 452. The majority (86.3%) of caregivers were 18–39 years old. ^a^ Reflects the number and percentage of participants answering ‘yes’ to this question

### Prevalence of common mental disorders (CMD)

To detect the group of caregivers who are at risk of developing common mental disorders (CMD), the SRQ-20 self-reported instrument was used [34] The SRQ-20 is a reliable and valid CMD measurement which consists of 20-item rating scale with a score range from 0 to 20 (the cut-off point ≥ 7). The results revealed that prevalence of CMD among caregivers (*N* = 450) in three study communities was 20.4% (*N* = 92) (Fig. 2).

Further t-test mean comparison analysis revealed significant differences between women and men *t*(61.05) = -2.41, *p* =.019. Specifically, women (*N* = 408; *M* = 4.22 (SD = 15.04)) reported significantly more mental health related symptoms (95%-CI[-2.57, -0.24]) than men (*N* = 42; *M* = 2.81 (SD = 3.42)).

### Factual and action knowledge about waste relationship to health and prevention

Only 28.3% (*N* = 130) of respondents answered that sanitary disposal of AHP means to ‘*dispose nappies in a designated bin/ separate plastic bag for nappies’* and 35.9% (*N* = 151) ‘*in a black plastic bag’* (Table 2).

**Table 2:** Factual and action knowledge about health risks and prevention

Question	Sample size *N* = 452	
	N	%
*What disadvantages are connected to NOT safely disposal of child nappies? Multiple answers*		
Unclean community	424	93.8%
Unhealthy environmental	415	91.8%
River pollution	404	89.4%
Spread of diseases	408	90.3%
*What reasons do you know that lead to getting sick? Multiple answers*		
Dirty hands	148	32.7%
Dirty surroundings	311	68.8%
Someone sick contagious / viruses	112	24.8%
Bacteria in general	98	21.7%
Eating unclean food	105	23.2%
Drinking unclean water	160	35.4%
Air pollution	143	31.6%
River pollution	118	26.1%
Unclean community/ environment	228	50.4%
Covid-19	40	8.8%
Burning trash/ nappies with chemicals	97	19.2%
*How can you prevent yourself and your children from getting sick? Multiple answers*		
Washing hands with soap	232	51.3%
Using a clean latrine	95	21.0%
Properly disposing of (child) nappies	201	44.5%
Only eating clean food	144	31.9%
Drinking safe/ clean water	183	40.5%
Keeping house and all appliances clean	204	45.1%
Keeping your community and river clean	186	41.2%
*Can you tell me what it means to dispose of children’s nappies in a safe way? Multiple answers*		
in garbage bin	226	50.0%
*in a designated bin/ separate plastic bag for nappies*	130	28.8%
into the toilet	133	29.4%
*in black plastic bag*	151	35.9%
burning	26	5.8%
throw in the forest	47	10.4%
dig the hole	58	12.8%
dump anywhere	16	3.5%
dump them at the sugarcane field	16	3.5%
burning with chemicals	20	4.4%
throw them at a nearby bush	8	1.9%

Note. *N* = 452. Scale: Yes/No/ I don’t know

### Use and sanitary disposal of AHPs

From 452 caregivers, 93.1% (*N* = 421) reported that they use child nappies. Only 18.4% (*N* = 83) of the respondents reported that in general they dispose AHPs in a designated/ separate plastic bag for nappies, and 58.4% (*N* = 264) in a black plastic bag. Furthermore, only 17.9% (*N* = 81) of respondents answered that last times they disposed AHPs ‘*in a designated bin/ separate plastic bag for nappies’*, however 49.8% (*N* = 225) disposed AHPs ‘*in a black plastic bag’* (Table 3).

**Table 3:** AHP (no) sanitary disposal practices (only users are presented)

Question	Sample size *N* = 421	
	N	%
*How do you dispose of your child’s nappies in general? Multiple answers possible*		
in garbage bin	125	27.7%
*in a designated bin/ separate plastic bag for nappies*	83	18.4%
into the toilet	48	10.6%
*in a black plastic bag*	264	58.4%
burning	42	9.3%
throw in the forest	56	12.4%
dig the hole	118	26.1%
dump any there	39	8.6%
dump them at the sugarcane field	27	6.0%
burning with chemicals	11	2.4%
throw them at a nearby bush	48	11.4%
*Last times, how did you dispose the nappies of your child? Multiple answers possible*		
in garbage bin	112	24.8%
*in a designated bin/ separate plastic bag for nappies*	81	17.9%
into the toilet	36	8.0%
*in a black plastic bag*	225	49.8%
burning	35	7.7%
throw in the forest	50	11.1%
dig the hole	100	22.1%
dump anywhere	33	7.3%
dump them at the sugarcane field	43	10.2%
burning with chemicals	14	3.1%
throw them at a nearby bush	31	6.9%
*What could you motivate to bring child nappies to the collection point? Multiple answers possible*		
Job creation for cleaning personal	360	79.6%
Regular collection (truck) of trash	43	10.2%
Recycling project	106	23.5%
Providing plastic bags	155	34.3%
Quick municipality response after reporting problem	152	33.6%
Unity and cooperation between community members	73	16.2%

Note. *N* = 421 (users)

### Self-reported behavioural frequencies

On average, interviewed users reported that they ‘quite = 3’ or ‘much = 4’ dispose sanitary child nappies ($$ M$$=3.83 (SD = 0.97)) on 5-point response scale from 1 (not at all) to 5 (very much). 3.1% of caregivers (*N* = 13) answered with ‘not at all = 1’, 6.9% with ‘a little = 2’ and 17.3% with ‘quite = 3’. In total, almost one third of caregivers do not dispose child nappies sanitarily (27.3% non-doers). Habitual behaviour to dispose child nappies sanitarily were reported by 67.5% of caregivers, but one third of caregivers reported that they do not dispose sanitarily child’s nappies as a matter of habit (without thinking) (32.5% non-doers). Most of them reported that they intend to dispose child nappies sanitarily (86.9%) and most of them intend to bring them to the collection point (84.6%) (Table 4).

**Table 4:** Behaviour, habit, and intention for sanitary disposal of AHPs frequencies and average

Question	Item	Doers %	Non-doers %	Average M (SD) %
Do you always dispose your child’s nappies in a safe way?	Behaviour 1 (sanitary disposal)	72.7	27.3	3.83 (0.97)
How often does it happen to you that you leave your child’s nappies on the ground without disposing of it?	Behaviour 2 (sanitary disposal, revers)	68.6	31.4	2.24 (1.05)
If there would be a collection point for child nappies in your community, would you bring child nappies to that point?	Behaviour 3 (collection)	-	-	70.8% (Yes)
How much do you feel that you dispose of your child’s nappies safely without thinking?	Habit (sanitary disposal)	67.5	32.5	3.72 (1.08)
How strongly do you intend to dispose of your child’s nappies safely?	Intention 1 (sanitary disposal)	86.9	13.1	4.25 (0.75)
How strongly do you intend to bring your child nappies to the collection point?	Intention 2 (collection)	84.6	15.4	4.24 (0.82)

Note. *N* = 421. Behaviour, habit, and intention to dispose sanitarily AHPs response scale: 1 = not at all, 2 = a little, 3 = quite, 4 = much, and 5 = very much’; 1= (almost) never, 2 = seldom, 3 = sometimes, 4 = often, 5=(almost) always. Doers = 4–5, non-doers = 1–3. Behaviour 3: yes/no

### Behavioural determinants

To investigate which psycho-social factors are determinants for sanitary disposal of AHPs among caregivers we used linear regression with sanitary disposal of AHPs behaviour as the dependent variable and the RANAS psycho-social factors as independent variables.

The regression analysis revealed that ten psycho-social factors significantly predicted sanitary disposal of AHPs: The model explained a variance of 45.6% in the sanitary disposal of AHPs behaviour (Table 5). A higher level of sanitary AHPs disposal was significantly related to *perceived vulnerability* (*β* = 0.239, *p* =.000), and *factual knowledge* about the links between health and waste (*β* =–0.086, *p* =.033). *Affective beliefs*, such as feeling proud, stress free, like, or happiness (*β* = 0.214, *p* =.010), *beliefs about prevention, safe and clean environmental* (*β* =–0.159, *p* =.010) connected to the sanitary disposal of AHPs also significantly predicted sanitary disposal of AHPs behaviour. Social norm (*personal obligation*) (*β* =–0.112, *p* =.049) significantly predicted higher frequency of sanitary disposal of AHPs as well. *Action knowledge* (how-to-do) (*β* =–0.187, *p* =.000), self-efficacy in a hurry, which represents *confidence in performance* (*β* = 0.142, *p* =.035), *recovering after disruption* (*β* = 0.171, *p* =.001), *action control/ planning* (*β* =–0.139, *p* =.001) and *remembering* to dispose sanitarily AHPs were significant predictors of target behavioural outcome (*β* = 0.302, *p* =.000).

**Table 5:** Behavioural determinants of sanitary disposal of AHPs

Psycho-social factor	B	β	t	*p*-Value
**Risk Factors**				
Perceived vulnerability 1 ***	0.239	0.219	4.116	0.000
Perceived vulnerability 2	0.049	0.058	1.128	0.260
Perceived severity	0.008	0.006	0.155	0.877
Factual knowledge (sum 0–22) *	–0.023	–0.086	–2.141	0.033
**Attitude Factors**				
Instrumental beliefs: time	–0.003	–0.004	–0.095	0.924
Instrumental beliefs: effort	0.80	0.068	1.353	0.177
Instrumental belief: Disease prevention, safe & clean environment**	–0.224	–0.159	–2.590	0.010
Affective beliefs negative (feelings) bad, uncomfortable, disappointed, disgusted	–0.147	–0.110	–1.374	0.170
Affective beliefs positive (feelings) ** happy, proud, like, stress free	0.298	0.214	2.576	0.010
**Norm Factors**				
Descriptive norm (family) Behaviour of others	–0.009	–0.010	–0.215	0.590
Descriptive norm (community) Behaviour of others	0.023	0.025	0.540	0.590
Injunctive norm (others approval)	–0.004	–0.003	–0.061	0.952
Personal norm (obligation)*	–0.131	–0.112	–1.972	0.049
Personal norm (respected person)	0.055	0.052	0.944	0.346
**Ability Factors**				
Action knowledge (how-to-do) ***	–0.388	–0.187	–4.553	0.000
Self-efficacy Confidence in performance	–0.063	–0.055	–0.851	0.395
Self-efficacy (hurry) * Confidence in performance hurry	0.142	0.142	2.114	0.035
Self-efficacy Confidence in continuation (barriers)	0.108	0.044	0.955	0.340
Self-efficacy (distance)	–0.120	–0.118	–1.629	0.104
Self-efficacy (no truck)	0.014	0.013	0.205	0.837
Self-efficacy Confidence in recovering (disruptions) ***	0.192	0.171	3.259	0.001
**Self-regulation Factors**				
Action control***	–0.129	–0.139	–3.504	0.001
Coping planning	0.208	0.080	1.747	0.081
Remembering***	0.338	0.302	4.914	0.000
Commitment	0.016	0.013	0.201	0.841

Note. *p ≤.05, **p ≤.01, ***p ≤.001. Adj. R^2^ = 0.456. *N* = 421; B = unstandardized beta value; *β* = standardised beta value; Behavioural question: *Do you always dispose your child’s nappies in a safe way?* All responses were recorded on 5-point response scales with choices from ‘1 - not at all’ to ‘5– very much’, 1= (almost) never, 2 = seldom, 3 = sometimes, 4 = often, 5=(almost) always. Coping plan and action knowledge scale: 0–1 (No/Yes); health knowledge: sum scale (0–22); action knowledge: sum scale (0–8); Mental health: sum scale [1–20]

These results suggest that by enhancing any of the ten significant psycho-social factors, while controlling for others (all other factors hold constant), an increase in the safe disposal of AHPs among caregivers can be expected. Specifically, an increase in the safe disposal of AHPs by 23.9% is anticipated among caregivers who recognize the health risks of unsafe AHP disposal (*perceived vulnerability 1*). Additionally, an increase in the safe disposal of AHPs is expected from 2.3% of caregivers who are aware of the links between waste and health (*factual knowledge*), 38.8% who understand how to safely dispose of AHPs (*action knowledge*), and 22.4% who believe that safe disposal of AHPs prevents diseases (*beliefs about prevention, safe and clean environmental*). A further increase by 29.8% is anticipated among those who experience positive feelings (*affective beliefs*), and approximately 13.1% among those who feel a personally obliged (*personal obligation*) to dispose of AHPs safely. Moreover, an improvement in the safe disposal of AHPs by 14.2% is likely among caregivers who are confident in their ability to correctly dispose of AHPs even in a hurry (*confidence in performance in a hurry*) and 19.2% among those confident in their ability to continue safe practices even when faced with obstacles (*confidence in recovery*). An increase by 12.9% is also expected among caregivers who are attentive (*action control*) and 33.8% by those who remember (*remembering*) to dispose of AHPs safely.

### Interaction effects between psycho-social factors and mental health on behavioural outcome

To investigate whether mental health influence the relationship between relevant psycho-social factors and sanitary disposal of AHPs, correlations (Spearman), and moderation analysis using PROCESS for SPSS 28 were applied [37]. Our moderation model included mental health as moderator (M), sanitary disposal of AHPs as outcome (Y), and psycho-social factors as predictors (X). Only significant psycho-social factors from linear regression analysis were tested.

To investigate the relationship between mental health and behavioural outcome, we used correlation analysis. The results revealed a significant positive relationship (Spearman correlation) between mental health and higher behavioural frequency to not dispose sanitarily AHPs (*r* =.099*). Caregivers with poor mental health were more likely to not dispose sanitarily AHPs.

Five moderation models showed significant interaction effects between mental health and psycho-social factors: positive feelings, perceived vulnerability, belief about prevention, safe & clean environment, confidence in performance, and remembering sanitary disposal of AHPs.

**Fig. 3:**
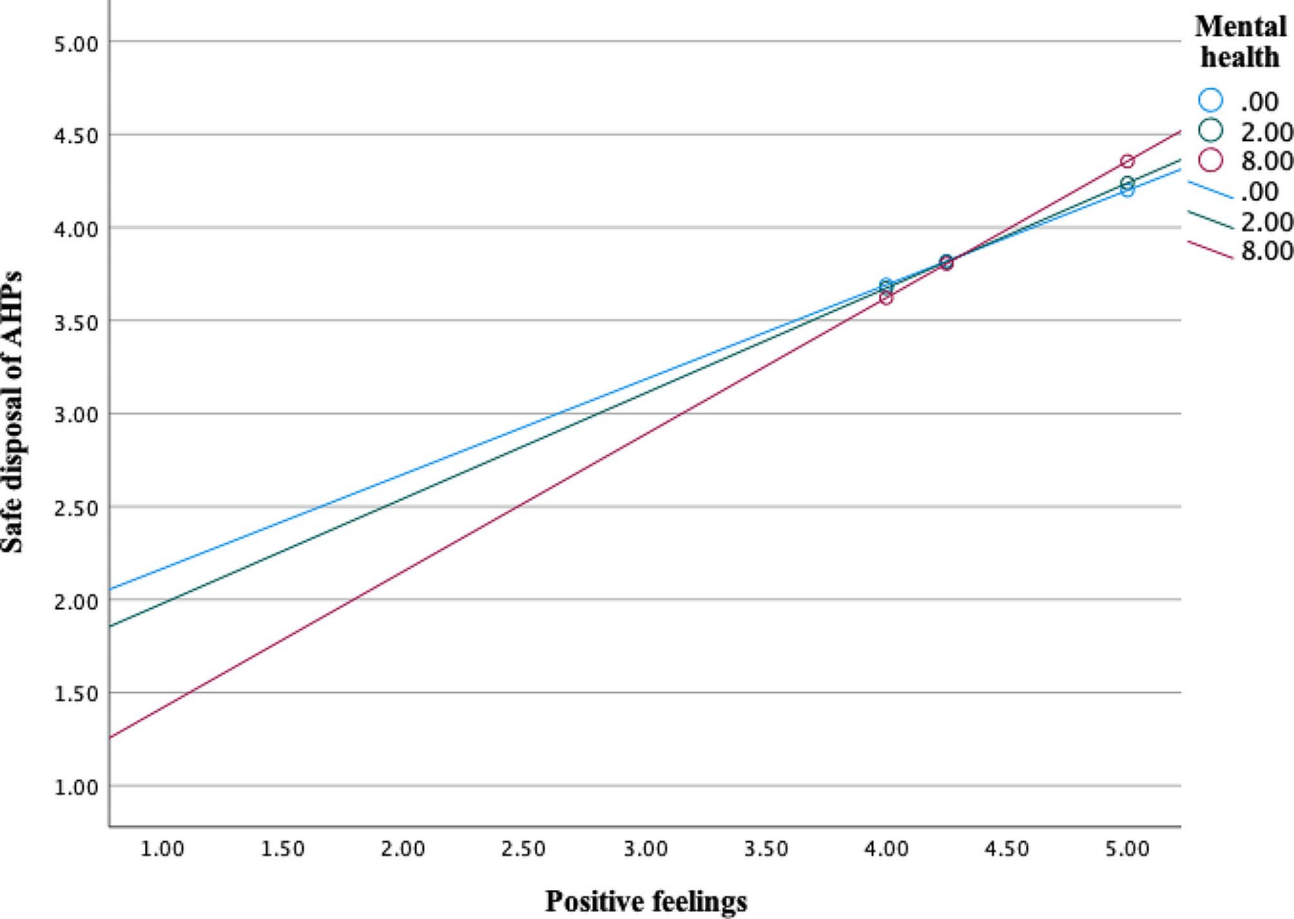
Interaction effects between mental health and psycho-social factor ‘positive feelings’ on self-reported sanitary disposal of AHPs. Mental health values are the 16th, 50th, and 84th percentiles (SRQ-20 scale 0–20)

Moderation analysis revealed significant interaction effects between mental health (M) and psycho-social factor *positive feelings* (X) on sanitary disposal of AHPs as an outcome (*b* = 0.0283, 95% CI [0.0056, 0.0510], t = 2.45, *p* =.0148). Mental health moderated the effects of psycho-social factor *positive feelings* on sanitary disposal of AHPs (Fig. 3).

**Fig. 4:**
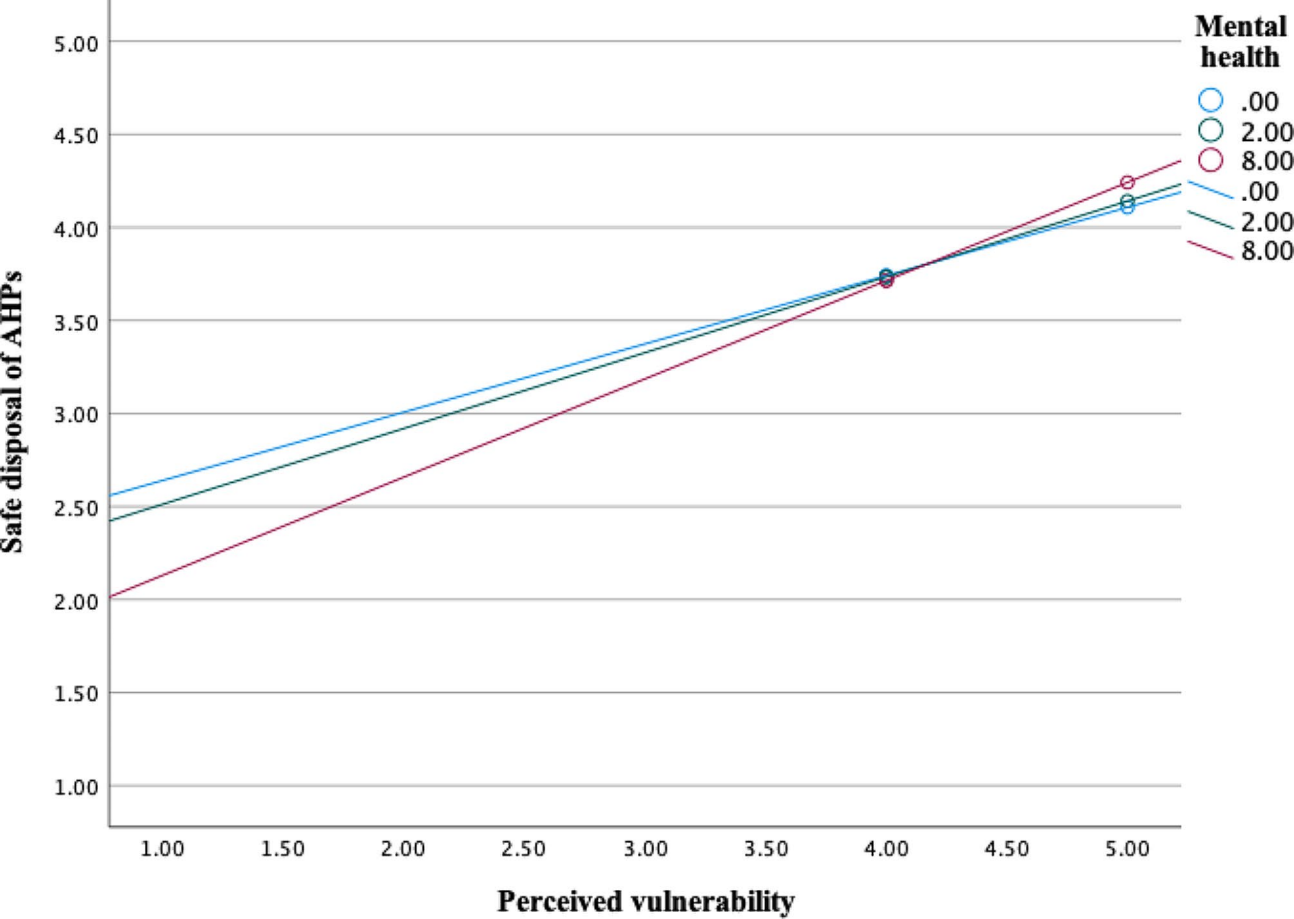
Interaction effects between mental health and psycho-social factor ‘perceived vulnerability’ on self-reported sanitary disposal of AHPs. Mental health values are the 16th, 50th, and 84th percentiles (SRQ-20 scale 0–20)

**Fig. 5:**
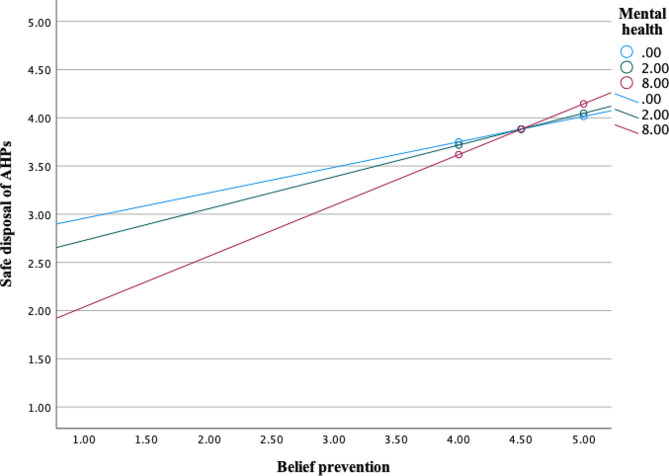
Interaction effects between mental health and psycho-social factor ‘belief prevention, safe & clean environment’ on self-reported sanitary disposal of AHPs. Mental health values are the 16th, 50th, and 84th percentiles (SRQ-20 scale 0–20)

**Fig. 6:**
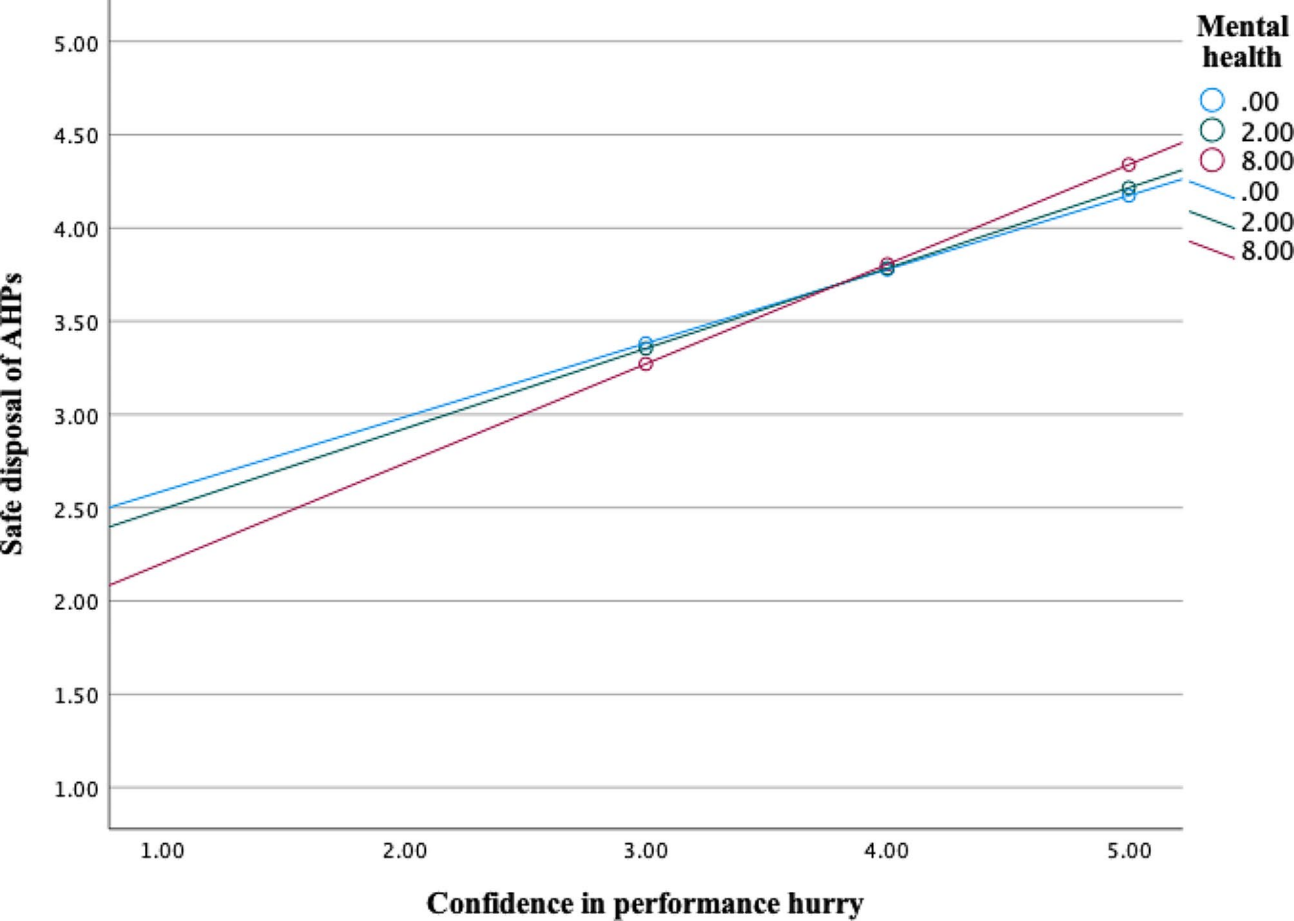
Interaction effects between mental health and psycho-social factor ‘confidence in performance in a hurry’ on self-reported sanitary disposal of AHPs. Mental health values are the 16th, 50th, and 84th percentiles (SRQ-20 scale 0–20)

**Fig. 7:**
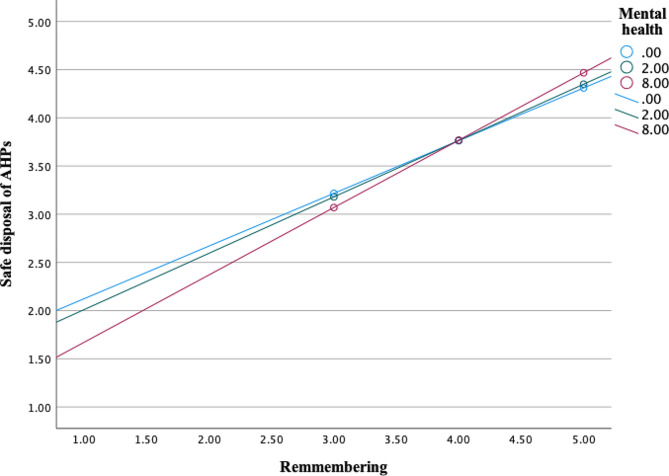
Interaction effects between mental health and psycho-social factor ‘remembering’ on self-reported sanitary disposal of AHPs. Mental health values are the 16th, 50th, and 84th percentiles (SRQ-20 scale 0–20)

Further, analysis showed significant interaction effects between mental health (M) and psycho-social factor *perceived vulnerability* (X) on sanitary disposal of AHPs as an outcome (*b* = 0.0201, 95% CI [0.0025, 0.0377], t = 2.25, *p* =.0250) (Fig. 4). Interaction effects were also significant in a moderation model with psycho-social factors *belief about prevention, safe & clean environment* (*b* = 0.0328, 95% CI [0.0058, 0.0598], t = 2.39, *p* =.0173) (Fig. 5), *confidence in performance in a hurry* (*b* = 0.0173, 95% CI [0.0010, 0.0336], t = 2.08, *p* =.0381) (Fig. 6) and *remembering* about sanitary disposal of AHPs (*b* = 0.0190, 95% CI [0.0016, 0.0365], t = 2.15, *p* =.0325) (Fig. 7).

In summary, the relationship between psycho-social factors positive feelings, perceived vulnerability, belief about prevention, safe & clean environment, confidence in performance (hurry), and remembering (X) and sanitary disposal of AHPs (Y) varied as a function of the mental state of the caregivers (M), meaning that the relationship depends on the mental state of caregivers. Though the relationship was positive, it was more positive among mothers and caregivers with good mental health.

## Discussion

### Interpretation of results

This study initiated an interdisciplinary exploration of psycho-social factors and underlying mechanisms, such as mental health, influencing caregivers’ behaviour regarding the collection and sanitary disposal of AHPs in three low-income communities within eThekwini Municipality, Durban, South Africa. By integrating approaches from psychology, geography, engineering, and economics, our research aimed not only to map the quantitative waste generation and dumping hotspots but also to develop and implement behaviour change (BC) intervention strategies for enhancing the sanitary disposal of AHPs and initiating an AHP collection and recycling pilot.

Our findings revealed a concerning trend: approximately one-third of caregivers do not practice sanitary disposal of child nappies, despite a high intent reported for future sanitary disposal (86.9%) and collection (84.6%) practices. This discrepancy underscores a gap between intention and behaviour, potentially exacerbated by socio-economic constraints and mental health challenges. Notably, the study highlights a significant prevalence (20.4%, every 5th caregiver was affected) of poor mental health among caregivers, with women being significantly more affected than men. This aligns with broader research indicating the negative impact of mental health on daily health-related behaviours, especially in vulnerable populations within low-income contexts. Comparatively, our findings resonate with studies from Malawi [21–23], yet they also underscore the critical need for targeted mental health interventions within BC strategies, a novel insight that adds depth to the existing literature on waste management and health behaviours.

Our application of the RANAS Model to explained a significant portion (45.6%) of the variance in sanitary disposal behaviours which reaffirms its utility across diverse contexts, particularly in low-income countries (see publications: https://www.ranasmosler.com/publications). The identification of key determinants provided a nuanced understanding of the behavioural ecosystem surrounding AHP disposal. The most important determinants of sanitary disposal and collection of AHPs were perceived vulnerability about personal health and environmental risks, health related factual knowledge, positive feelings towards sanitary disposal and collection of AHPs, beliefs about prevention, safe and clean environment, personal obligation, action knowledge (how-to-do), confidence in performance (hurry and recovering), action control/planning and remembering of sanitary disposal and collection of AHPs. Consequently, by targeting those psycho-social factors with BC interventions we expect higher frequencies of sanitary disposal of AHPs among mothers and caregivers after the intervention. This interdisciplinary analysis not only validates previous findings but also reveals psycho-social factors that can inform more effective BC interventions.

By analysing the role of mental health, our study contributes fresh insights into the moderating effects of mental health on environmental and health behaviours, advocating for a more holistic approach to intervention design. Our study results are in line with previous research that mental health moderates the effects of several psycho-social factors on target behaviour [21–23]. That is, the prevalence of targeted behaviour depends on the mental state of caregivers. While this relationship was positive for all participants, it was more positive among mothers and caregivers with better mental health. The pronounced impact of mental health on sanitary disposal behaviours underscores an urgent need for integrated BC strategies that address mental health, particularly among women. Our findings suggest that targeting psycho-social factors, including health knowledge, environmental beliefs, and personal obligations, could significantly enhance sanitary disposal practices. However, the integration of mental health interventions presents a novel pathway to bolstering these efforts, potentially offering a blueprint for similar initiatives globally.

In summary, our investigation extends beyond the mere quantification of waste and mapping of dumping hotspots to uncover the deeply entrenched psycho-social and mental health factors influencing AHP disposal behaviours in low-income communities. By highlighting the critical role of mental health and providing a comprehensive analysis of behavioural determinants, our study not only corroborates existing research but also charts new directions for future studies and policy. The insights derived from this interdisciplinary effort offer a valuable contribution to the ongoing discourse on sustainable waste management, mental health, and community resilience, steering towards more informed and effective behaviour change interventions.

### Limitations

The study’s communities were chosen through purposive sampling, focusing on three specific communities in relation to the city of Durban. Consequently, the insights obtained are closely linked to these communities, limiting the generalizability of our conclusions across different South African regions or other socio-economic contexts. This sampling approach, while beneficial for in-depth, context-specific understanding, may not reflect the full spectrum of experiences and behaviours present in varied settings.

Furthermore, the study’s emphasis on psycho-social factors, though comprehensive, might not have captured all potential variables influencing the sanitary disposal of AHPs. The complex interplay of economic, cultural, and infrastructural factors also deserves attention, as these could significantly affect the implementation and effectiveness of BC interventions in diverse communities.

Future research should consider expanding the geographic scope of study to include a wider range of communities, utilizing random sampling methods where feasible to enhance the representativeness of the findings. Additionally, investigations into the role of economic and cultural factors, alongside the psycho-social determinants explored in this study, could offer a more holistic view of the barriers and facilitators to sanitary AHP disposal. Implementing longitudinal studies could also reveal insights into the long-term effectiveness of BC interventions and the sustainability of behaviour change over time.

By addressing these limitations and following the outlined future directions, subsequent research can build upon our findings, offering deeper insights and more robust recommendations for improving waste management practices, mental health, and community resilience across varying contexts.

### Practical implications

The study underscores the need for a comprehensive intervention strategy targeting critical psycho-social factors. Additionally, the intervention should leverage the most trusted communication sources identified by participants, including family, friends, and local media, to effectively disseminate behaviour change messages. Moreover, the successful implementation of behaviour change strategies necessitates not only tailored communication but also the provision of essential infrastructure, such as bins and collection systems, and the transformation of dumpsites into community spaces, thereby fostering a holistic approach to promoting sanitary disposal practices. Furthermore, addressing mental health is crucial, recognizing that the psychological well-being of caregivers involved is essential for the sustained success of these practices.

#### BC intervention strategy for sanitary disposal and collection of AHPs

The study results (Table A1 in Annex) revealed that an intervention strategy should target the following psycho-social factors: *perceived vulnerability*, *factual knowledge* about relationship between health and waste, *beliefs about prevention, safe & clean environment*, affective beliefs (*positive feelings*), social norm (*personal obligation*), *action knowledge*, self-efficacy (*confidence in performance and recovery*), *action control*, and remembering (Table A1 in Annex). Behaviour change techniques (BCT’s) are selected from the RANAS BCT’s catalogue^1^. Intervention strategy should be discussed during the two days BC intervention development workshop with key stakeholders about the feasibility of BC interventions and inform the final BC intervention implementation guide. Intervention strategy includes targeted psycho-social factors, behaviour change techniques (BCT’s), specific activities and messages, communication channels (Table A1 in Annex) and the most trusted communication sources (Table A2 in Annex).

Additional to evidence-based BC strategy, infrastructure such as bin and collection system should be provided. Furthermore, the dump sides should be cleaned before the intervention and community- based incentives (i.e. transformation of the dump sides into green spaces) should be implemented parallel to or after BC strategy implementation.

#### Mental Health intervention

The study results indicate the importance of addressing mental health among caregivers for the effective implementation of sanitary disposal practices. As a practical implication, the Problem Management Plus (PM+) program [38], suggested by the WHO, offers a feasible and scalable solution to address the acute shortage of mental health services in low- and middle-income countries (LMICs). PM + is a low-intensity, transdiagnostic psychological intervention that can be delivered by trained lay helpers, effectively bypassing the barriers of limited funding, insufficient infrastructure, and the scarcity of mental health professionals in these regions. By focusing on core strategies like stress management, problem-solving, behavioural activation, and strengthening social support, PM + addresses a wide range of common mental health issues, making it a versatile tool in diverse cultural settings. The implementation of PM + as a community-based intervention aligns with the need for accessible, cost-effective, and culturally sensitive mental health solutions, promising to significantly enhance mental health care delivery and outcomes in LMICs.

## Conclusions

This was the study investigating psycho-social factors and underlying mechanisms (i.e. mental health) related to sanitary disposal and collection of AHPs among mothers and caregivers in low-income and informal communities in Durban, South Africa. Our research findings confirmed the link between poor mental health and unsanitary AHPs disposal. This is especially relevant because poor mental health is common within South Africa. Addressing mental health problems within these communities is an essential step to providing sustainable waste management services. The impact of these interventions will lead to a cleaner environment and better health and mental health among community members. Our research findings are an important contribution to the long-term strategy of achieving the Sustainable Development Goals (SDGs) and contribute to the inclusion of vulnerable caregivers with poor health and mental health living in low-income communities, in humanitarian action related to environmental and climate change through evidence-based BC intervention implementation.

### Electronic supplementary material

Below is the link to the electronic supplementary material.

Supplementary Material 1

## Data Availability

The dataset generated and analysed during the current study are available upon reasonable request. Interested researchers would contact the corresponding author at mkalina@ethz.ch for access.

## Footnotes

1 www.ranasmosler.com.
