# Supplementary material for: Absorbent hygiene products disposal behaviour in informal settlements: identifying determinants and underlying mechanisms in Durban, South Africa
Source: BMC Public Health. 2024 Mar 28;24:912. doi: 10.1186/s12889-024-18396-y (PMC10976708; doi:10.1186/s12889-024-18396-y)
Supplement: Supplementary file 1 — Supplementary Material 1 [file 12889_2024_18396_MOESM1_ESM.docx]

**Table A1** Intervention strategy for sanitary disposal of child nappies (AHPs)

|  | Targeted psycho-social factor and result  Caregivers whose average behaviour to dispose child nappies sanitary is lower… | Behaviour change technique (BCT) | Activities and  messages | Communi-cation  channel |
| --- | --- | --- | --- | --- |
| Information BCTs | **Perceived vulnerability**  … perceive *themselves* less *at risk to get a disease through waste* | *BCT 3: Inform about and assess personal risk* | Present qualitative and quantitative assessments individually for each person in such a way that the person realises about advantages of sanitary disposal of AHPs | Caregivers meeting, household visit, video TV spots |
|  | **Factual knowledge**  … know less about *health-related risks and links* | *BCT 1: Present facts* | Present information about the relationship of health and waste | Posters, flyers |
| Persuasive BCTs | **Affective beliefs (feeling *proud*, *happy*, *stress free*)**  … feel less *proud*, happy, and *stress free* when *dispose AHPs sanitary* | *BCT 8: Describe feelings about performing and consequences of the behaviour* | Present the performance and the consequences of a sanitary disposal of AHPs as pleasant and joyful and its omission. The participant can be invited to assess and describe his/her feelings by him/herself, others can testify their feelings or feelings can be induced. | Caregivers’ meetings, video spots, posters with happy mothers |
|  | **Beliefs about prevention, safe & clean environment**  … believe less that sanitary disposal of AHPs prevents diseases and creates safe & clean environment | *BCT 6: Use subsequent reward*  *BCT 7: Prompt to talk to others* | Reward the person each time she/he has performed the desired behaviour or achieved the behavioural outcome.  Invite participants to talk to others about the healthy behaviour in question. | Caregivers meeting, messaging WhatsApp, text |
| Normative BCTs | **Personal obligation**  …feel only somewhat *obligated to dispose AHPs sanitary* | *BCT 13: Provide a positive group identity*  *BCT 12: Prompt anticipated regret* | Describe people already engaged in the behaviour in an attractive way, e.g. as modern and up-to-date, to view the proposed behaviour change as having a positive influence on one’s identity. People may aspire to be one of those positively described persons and therefore change behaviour/intention.  Bring people to imagine the concerns and regret they would feel after performing undesired behaviours which are not consistent with their personal norms of living healthily and caring for their children. | Posters with happy mothers and children,  Text/  WhatsApp messages |
| Infrastruc-tural, skill & ability BCTs | **Action knowledge (how to do)** … don’t know how to dispose AHPs sanitarily | *BCT 15: Provide instruction*  *BCT 16: Provide infrastructure*  *BCT 20: Facilitate resources* | Convey know-how to improve a person’s knowledge about how to perform the respective behaviour. What does it mean to dispose AHPs sanitarily?  Prompt and support the community or households to set up infrastructure.  Provide financial help. It may be unconditional or conditional, meaning the recipient has to contribute (e.g. with manpower) to get the resources. | Caregivers’ meetings, household visits, flyers |
|  | **Self-efficacy (confidence in recovering)**  … feel only somewhat confident that they can sanitarily dispose AHPs when they were disrupted | *BCT 25: Prompt coping with relapse* | Tell participants that lapses are normal when recovering, adopting a new behaviour and, though discouraging, not a sign of failure. |  |
|  | **Self-efficacy (hurry/ coping with barriers)**  … feel only somewhat that they can overcome barriers regarding *sanitarily disposal of AHPs (hurry)* | *BCT 24: Reattribute past successes and failures* | Prompt participants to attribute successes as personal achievements and failures to adverse circumstances or to not yet developed but achievable skills instead of to personal deficiency. |  |
| Planning & relapse prevention BCTs | **Action Control**  …pay less attention to dispose AHPs sanitarily  **Remembering**  Remember less to dispose AHPs sanitarily… | *BCT 28: Provide feedback on performance*  *BCT 34: Use memory aids & environmental prompts* | Give participants feedback on their behaviour performance.  Households are encouraged to hang up a poster with a reminder to dispose AHPs sanitarily in a visible place in a house (a sticker with a text, picture or painting representing sanitarily disposal of AHPs). | Text/  WhatsApp messages, reminders (stickers),  posters |
| Mental health | *n/a* | *n/a* | Specific intervention targeting mental health of mothers | Caregivers group meetings; individual sessions |

**Table A2** Trusted communication sources (percentages of yes, multiple answer possibilities)

| Source | Percentages (Yes) |
| --- | --- |
| Family | 37.4 |
| Friends | 29.6 |
| Neighbour(s) | 29.4 |
| Community centre | 22.3 |
| Teacher | 11.1 |
| Authority figure (e.g. political person / admin / mayor) | 16.4 |
| Health-social-workers/ doctor/ nurse | 13.9 |
| Health clinics | 21.7 |
| Children | 4.2 |
| Social media (facebook, instagram, youtube) | 15.3 |
| Newspapers | 19.5 |
| Radio | 17.3 |
| TV | 25.7 |
| Internet (google) | 10.4 |

*Note: N=*421.
